# The Ear: Its Anatomy, Physiology and Diseases

**Published:** 1884-11

**Authors:** 


**The Ear: Its Anatomy, Physiology and Diseases.** A practical treatise for the use of medical students and practitioners. By CHARLES H. BURNETT, A. M., M. D., Professor of Otology in the Philadelphia Polyclinic and College for Graduates in Medicine. With one hundred and seven illustrations. Second edition, revised and rewritten. Philadelphia: Henry C. Lea's Son & Co. 1884.

The second edition of this excellent work on otology embodies the advances made in this important department up to the present time. The author brings to the work which he issues to the profession a wide experience and a ripe culture, both literary and professional. The result of these labors is certainly a credit to American medical literature. The publishers have liberally supplemented the efforts of the author with accurate illustrations and clear typography, on a superior quality of paper, so that the volume is among the best we have had presented for examination and review. The contents show the care with which the author has revised the work. Many chapters of the first edition have been entirely rewritten, among which may be enumerated the abnormalities of the auricle, otomycosis, the treatment of chronic otorrhœa, the classification and treatment of aural polypi, and the diagnosis, etiology and treatment of aural vertigo. Obsolete matter has also been omitted, and the valuable work offered in a volume of 585 pages. When one considers the frequency of aural diseases, and the tendency to deafness often resulting therefrom, the value of a comprehensive work devoted exclusively to their consideration will be appreciated by the profession outside of the specialists. We commend this work to the profession.

